# Bilateral thoracic trauma; presentation and management, a case series

**DOI:** 10.1016/j.amsu.2019.05.006

**Published:** 2019-05-25

**Authors:** Aram Baram, Fahmi H. kakamad

**Affiliations:** a Faculty of Medical Sciences, School of Medicine, Department Cardiothoracic and Vascular Surgery, University of Sulaimani, François Mitterrand Street, Sulaimani, Kurdistan Region, Iraq; b Kscien Organization for Scientific Research, Hamdi Street, Sulaimani, Kurdistan, Region, Iraq

**Keywords:** Bilateral thoracic trauma, Mortality, Management, Outcome

## Abstract

**Introduction:**

Unilateral chest trauma has been perfectly described in the literature while bilateral chest trauma has never been specifically probed, the aim of this study is to highlight the specificities, presentations, the difference in the therapeutic algorithm and outcome of patients with bilateral thoracic trauma.

**Patients and methods:**

A single center, prospective study was carried out in four years. The data were taken directly from the patients, patient's relatives and the medical records. All patients presenting with bilateral chest trauma, admitted to the hospital overnight, were included in this study. The patients were managed according to the Advanced Trauma Life Support (ATLS) protocol which consists of primary and secondary surveys. For those patients who diagnosed to have either haemo or pneumothorax or both, thoracostomy tube was inserted. Descriptive and analytical analyses were calculated.

**Results:**

The study included 107 patients. Bilateral blunt trauma was found in 72 (67.3%) cases while bilateral penetrating trauma was found in 35 (32.7%) patients. The most common mechanism of trauma was road traffic accidents (RTA) accounting for 68 (63.6%) victims. Overall 30-day mortality was 14.9%. In blunt trauma, 3 or more rib fracture, pulmonary contusion, intubation, and intensive care unit admission were among the predictors of increased risk of mortality.

**Conclusion:**

Bilateral thoracic trauma has comparable patterns of presentation, choices of investigation, strategies of management, predictors of the outcome, morbidity and mortality with unilateral chest trauma.

## Highlights

• Unilateral chest trauma has been perfectly described in the literature.
• While bilateral chest trauma has never been specifically probed in the literature.
• This study highlights the details of bilateral thoracic trauma.

## 1 Introduction

Trauma is regarded as the most common cause of death in young and middle age groups [1]. The most frequent body parts involved in serious injuries are the cranium (43%), chest (28%) and abdomen (19%) [2]. Rib fracture is the most common skeletal insult which can be found in 50% of cases with blunt chest trauma [3]. During the first hour in the hospital, the most common causes of death are thoracic and central nervous system injuries [2]. Despite the improved methods of management, chest trauma accounts for 25% of trauma deaths and it plays a major role in as many as a further 50% of in-hospital mortality [2,4]. The morbidity and mortality of trauma patients are significantly affected by the coincidence of chest trauma, which may result in pulmonary capillary leakage syndrome with subsequent interstitial edema, loss of compliance, ventilation/perfusion mismatching, intra-bronchial and intra-alveolar bleeding and an enhanced systemic inflammatory response [4,5]. In general, victims of thoracic trauma have about 10% mortality rate [2]. Mortality has been reported to be increased three folds (36.3%) when first rib fracture occurs [3]. From the pathophysiological point of view and impact of the injuries, chest trauma classically has been divided into penetrating and blunt chest trauma. The overall mortality rate of the former is 11.5% [2,6]. Blunt trauma has more variable clinical courses with different mortality rates in various reports ranging from 3% to 25% according to the severity of the trauma and the magnitude of the insult [6,7]. In spite of this, classification of chest trauma into unilateral and bilateral injuries has never been probed in the literature. The impact of the latter on the general health, its risks, strategies of management and outcome are not well understood. The aim of this study is to highlight the presentation, management and outcome of patients presenting with bilateral thoracic trauma.

## 2 Methods

### 2.1 Registration

The research registry record has been captured in accordance with the declaration of Helsinki – “Every research study involving human subjects must be registered in a publicly accessible database before recruitment of the first subject”. The study was registered in Chinese Clinical Trial Registry. The registration number was ChiCTR1900021262.

### 2.2 Study design

This is a single center prospective case series study. The participants were consecutive in order. The paper has been written in line with the PROCESS criteria [8].

### 2.3 Setting

The patients were managed in an academic practice setting which is located in Kurdistan-Iraq. The study lasted four years from 1/10/2015 to 1/10/2018. The data were taken directly from the patients, patient's relatives and the medical records. The patients were followed up for an average of two years ranging from 3 months to 4 years. Approval has been granted from the Ethical and Scientific Council of Kurdistan Board for Medical Specialties (KBMS).

#### 2.3.1 Participants

All Patients presenting with bilateral chest trauma, admitted to the hospital overnight, were included in this study. The followings have been precluded from the study: (1) Any patient without bilateral chest trauma. (2) Patient with bilateral chest trauma but for which not admitted to the hospital. (3) Patient with bilateral chest trauma admitted to hospital but discharged within the 24 hours of admission.

#### 2.3.2 Work up and management

The patients were managed according to the Advanced Trauma Life Support (ATLS) protocol consisting of primary and secondary surveys commencing from the airway, breathing, circulation, exposure. Chest-x-ray and eFAST were requested for every patient after stabilization. Chest Computed Tomography scan (CT scan) was taken for patients with suspected major intra-thoracic injuries and those cases with findings unexplained by the chest-x-ray and eFAST. For the patients suspected to have plural collections by clinical examination and/or radiological imaging, appropriate thoracostomy tube was inserted under local anesthesia in the safety triangle.

#### 2.3.3 Who performed the procedures

A team consisting of a specialist, senior house office and nurses performed all the procedure. The first author was the team leader.

### 2.4 Post-intervention considerations

The patients were kept on intravenous antibiotics (1 g ceftriaxone 1 × 2), analgesic (paracetol bottle 1 × 2) and expectorant (solvodin ampule, 4 mg, 1 × 3) during the hospital stay, while they were advised to receive oral antibiotics (Ciprofloxacin 500 mg x 2) and analgesic (paracetol tablet 500 mg, 1 × 4) at least for the five days following discharge. The patients were followed up in a private clinic.

#### 2.4.1 Statistical analysis

The data were collected and entered into an excel sheet, after coding, they were transferred to the Statistical Package for the Social Sciences (SPSS) software, version 22. Descriptive and analytical analyses were calculated. Relationship between the initial findings and subsequent morbidity and mortality were found using chi square test for nominal variables and T-test for quantitative variables.

## 3 Results

The study included 107 patients. Seventy-three (68.2%) patients were male, 34 (31.8%) cases were female. The mean age was 34.98 years with a standard deviation of ±15.81 years, ranging from 7 to 74 years. Blunt trauma was found in 72 (67.3%) cases while penetrating trauma was found in 35 (32.7%) patients, Table 1.

**Table 1:** Basic characteristics of the patients according to the type of the trauma with comparison.

Variables	Blunt chest trauma	Penetrating chest trauma	p-values
Numbers (%)	72 (67.3)	35 (32.7)	N/A
Mean age (±standard deviation)	38.26 ± 17.6	28.23 ± 7.9	<0.001
Gender, n. (%)			
Male	42 (58.3)	31 (88.6)	0.02
Female	30 (41.7)	4 (11.4)	
Smokers: numbers (%)	21 (30)	29 (83)	<0.001
Past medical history			
-Diabetes	6 (8%)	1 (3%)	<0.001
-COPD	9 (12%)	4 (11%)	0.2
Presence of Tattoos	5 (6.9)	22 (62.9)	<0.001

N/A: not applicable.

COPD: chronic obstructive airway disease.

The most common mechanism of trauma was RTA accounting for 68 (63.6%) victims (94% of the blunt thoracic trauma), followed by stab wounds (30, 28%), bullet injuries (5, 4.6%) and fall from height (FFH) (4, 3.7%).

**Fig. 1:**
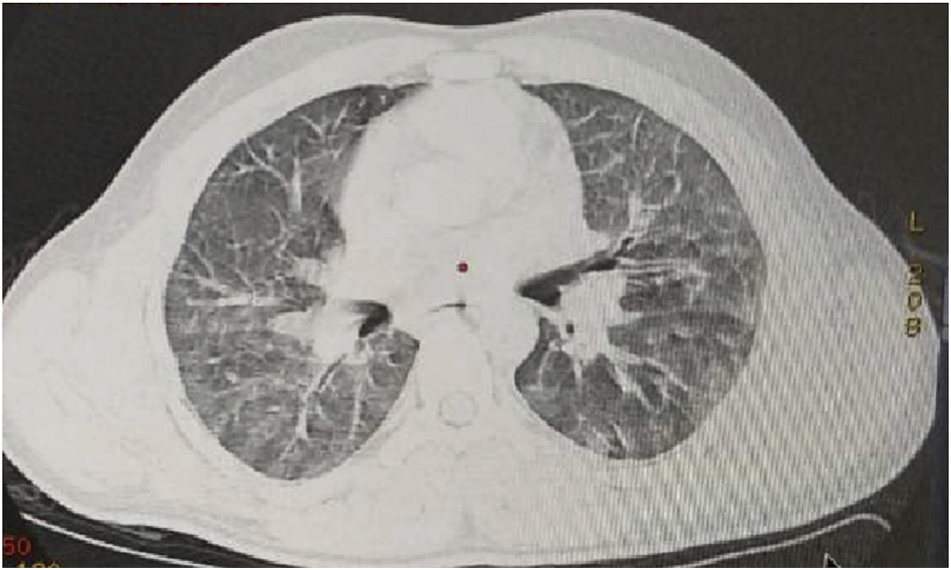
Computed tomography scan of a 25-year-old male subjected to blunt chest trauma revealing bilateral pulmonary contusion.

**Fig. 2:**
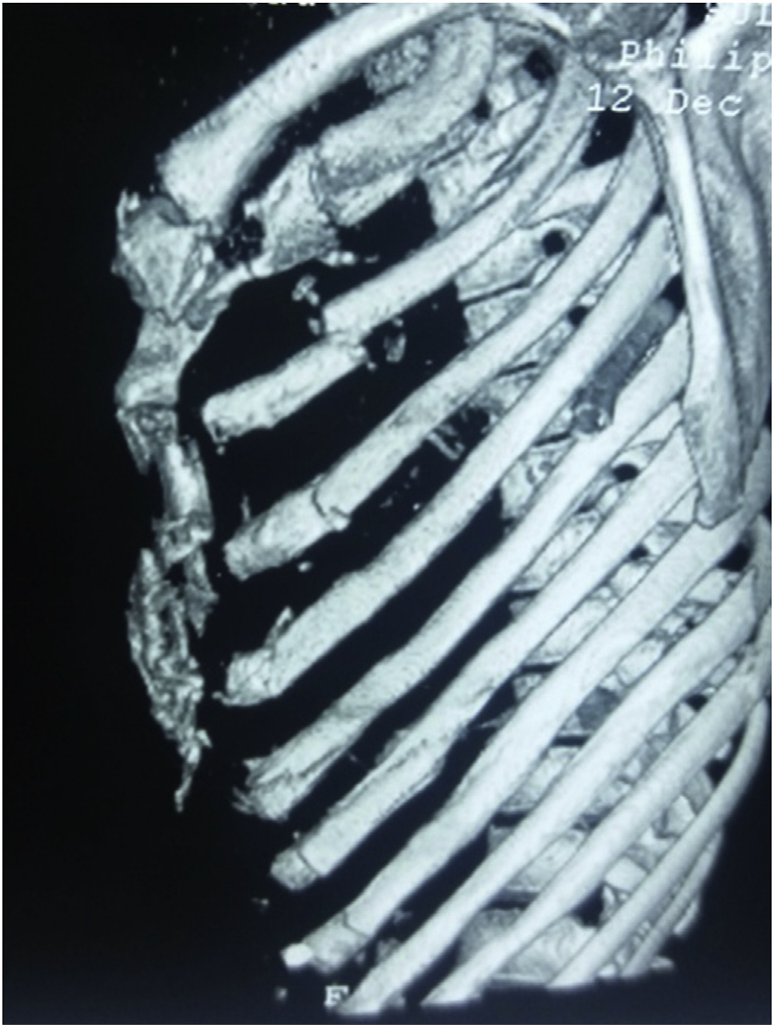
Three-D reconstructed computed tomography scan of a young female presented with blunt chest trauma demonstrating multiple rib fractures with sternal fracture.

Findings varied between the blunt and penetrating thoracic trauma. Pneumomediastinum occurred in 6 (5.6%) cases while pulmonary contusion was more common and it was found in 23 (21.5%) victims (Fig. 1). Multiple rib fractures were observed in 66 (91.7%) cases (less than 3 ribs in 12 cases, 3–6 ribs in 27 cases, more than 6 ribs in 21 cases, associated sternal fracture in 3 cases and flail segment in 3 cases) (Fig. 2). All of these three findings (pneumomediastinum, contusion and rib fracture) were exclusive to the blunt chest trauma (Table 2).

**Table 2:** Findings in both bilateral blunt and penetrating trauma with comparison.

Variables	Blunt trauma	Penetrating trauma	P-value
Plural collection			
**-**Pneumothorax no. (%)	26 (36.1)	17 (48.9)	
-Hemothorax no. (%)	26(36.1)	8 (22.9)	
-Hemopneumothorax,no. (%)	20 (27.8)	10 (28.6)	0.33
Presentation			
**-**Dyspnea	53 (73.6)	29 (82.9)	
- Associated shock	8 (11.1)	5 (14.3)	
-Associated LOC	8 (11.1)	1 (2.9)	
-Associated shock and LOC	3 (4.2)	0 (0)	0.281
Multiple rib fracture	66 (91.7)	0 (0)	N/A
Subcutaneous emphysema	62 (92.5)	5 (14.3%)	<0.001
Pneumomediastinum, n.(%)	6 (8.4)	0 (0)	N/A
Pulmonary contusion, n.(%)	23 (31.9%)	0 (0)	N/A
Associated extrathoracic injuries:			
-Vertebral fracture	8 (11.1%)	0 (0)	
- Intracranial hemorrhage	8 (11.1%)	0 (0)	
- Basal skull fracture	6 (8.3%)	0 (0)	
- Femur fracture	3 (4.2)	0 (0)	
- Abdominal organ injuries	1 (1)	4 (11.4%)	<0.001
Hospital stay (mean ± standard deviation) days	9.14 ± 4.24	6.83 ± 2.26	<0.001
Dead	15 (20.8)	1 (2.8)	0.014

LOC: loss of consciousness.

N/A: not applicable.

One hundred and six patients (99.1%) underwent both chest-x-ray and eFAST. One patient (0.9%) died during the resuscitation precluding any sort of investigation. He was a 26-year-old male presented with the disturbed level of consciousness (Glasgow Coma Scale 4), multiple stab wounds to the both anterior chest wall. Myocardium was exposed. Resuscitation was performed including direct cardiac massage without response. Fifty-three (49.5%) patients had a chest CT scan. Overall 30-day mortality was 14.9% (16 cases died). The disturbed level of consciousness on presentation was the most frequent finding among the patients who died 30 days within the insult (10 (62.5%) in the dead group versus 2 (2.2%) in the alive group, P-value <0.001) (Table 3).

**Table 3:** Predictors of mortality in bilateral chest trauma.

Variables	Survived (91 cases)	Deceased (16 cases)	P-value
Age (mean ± standard deviation)	34.1 ± 15.17	40.5 ± 18.62	0.131
Male no.(%)	66 (72.5)	9 (56.3)	0.190
Type of injury no.(%)			
-Penetrating trauma	34 (37.4)	1 (6.3)	0.014
-Blunt trauma	57 (62.7)	15 (93.8)	
Disturbed level of consciousness	2 (2.2%)	10 (62.5%)	<0.001
Subcutaneous emphysema no.(%)	55 (60.4)	12 (75.0%)	0.267
Pneumomediastinum no.(%)	5 (5.5%)	8 (50%)	<0.001
Pulmonary contusion no.(%)	8 (8.8%)	15 (93.8%)	<0.001
Rib fracture no.(%)			
1-3	9 (9.9)	2 (12.5)	0.017
3-6	23 (25.3)	5 (31.3)	
More than 6	16 (17.6)	5 (31.3)	
Plural collections			
-Hemothorax	27 (29.7%)	7 (43.8)	0.482
-Pneumothorax	36 (40.7)	6 (37.5)	
-Hemopneumothorax	27 (29.7)	3 (18.8)	
ICU admission	8 (8.8)	13 (81.3)	<0.001
Mechanical ventilation no. (%)	7 (7.7)	13 (81.3)	<0.001

ICU: intensive care unit.

## 4 Discussion

Chest trauma is regarded as an influential source of morbidity and mortality in various age groups [7,9,10]. Among 22613 cases of blunt chest trauma, Huber and colleagues reported a mean age of 46.1 years [10]. In the series of Cho et al., which included 130 cases of severe chest trauma, the mean age at the time of the presentation was around 50 years [3]. Even in the same study and the same geographical region with two different time periods, different age distribution has been reported. Edegbye and associates analyzed their 24-year registry of blunt chest trauma, they divided the participants into two time period, the first and the second dozens of years. The mean age of the cases in the first time period was 38.3 years while it was 56.4 years in the second half [9]. Regarding this report of bilateral chest trauma, the mean age was near 35 years, being older significantly in the blunt chest trauma (38.26 ± 17.6 years) and younger in the penetrating chest trauma (28.23 ± 7.9), p-value <0.001.

According to most of the reports, blunt thoracic trauma occurs more prevailingly and has a higher mortality rate than penetrating thoracic trauma. Penetrating thoracic trauma generally forms only one-third of the chest trauma patients [11]. In this regard, bilateral chest trauma may have the same picture as in the current study, two-third of the patients had blunt thoracic trauma and it is significantly associated with a higher risk of mortality (P-value 0.014).

A tattoo is a specific design drawn by inducing an exogenous dye or pigment into the dermis. Worldwide, in the recent few years, the number of persons having body tattoos has increased. Researchers think that it is more than merely a cultural and/or cosmetic habit. It may be a symbol for social rank, or member of a criminal and noncriminal groups, narrates an event, or reveals a personality [12]. Surprisingly, in this study, the number of patients having body tattoos was significantly higher in the penetrating trauma group than the blunt trauma groups (P-value <0.001). To best of our knowledge, there is no report supporting or rejecting this finding. Further studies are crucial to explain this result.

The most prevalent mechanism of thoracic trauma in this study was RTA, accounting for more than half of the total cases (63.6%) and 94% of the blunt chest trauma, this is in accordance with the international standard for the unilateral chest trauma [11,[13], [14], [15], [16]]. Rib fracture is a frequent manifestation of blunt chest trauma. It comprises 10% of all trauma admission [14,16,17]. It is considered by some studies as a risk factor for increased mortality while others denied this correlation [3,[18], [19], [20], [21]]. Analysis of a registry by Lien and colleagues which included 18856 patients with rib fracture showed that patients with six or more rib fracture were three times more in risk to die within 24 hours of hospitalization [22]. In the systematic review of Battle and associates, at least there were four studies which denied the relationship between rib fracture and increased risk of mortality [23]. In the current study, the rate of mortality significantly increased when there was ≥3 rib fracture.

Pulmonary contusion is another common finding in blunt chest trauma reaching about 30–70% in some reports. It is considered a predictor for the higher morbidity and mortality. This is because of impaired gas exchange, and development of acute respiratory distress syndrome due to systemic and local activation of the immune response [24,25]. The rate of pulmonary contusion found in this report (31.9% of the blunt trauma cases) is relatively lower than the international standard [9,24]. This may be explained by the fact that all patients in this study did not undergo chest CT scan and pulmonary contusion might be missed by plain chest-X-ray [25].

Pneumomediastinum which is sometime called mediastinal emphysema is defined as a collection of air surrounding the mediastinal contents. In less than 2% of the cases, the etiologies are related to the tracheaobronchial injures and very rarely due to the esophageal injuries while most of the time, the causes and the mechanisms are not known. Macklin effect is a three-step pathophysiological process which was proposed to explain pneumomediastinum in those cases without aero-digestive tract injuries. It includes first, pulmonary contusion with alveolar rupture, second, dissection of the air along the bronchovascular sheath, and lastly, spreading of the air into the mediastinum. However, macklin effect was only observed in 37% of the cases with mediastinal emphysema. Following blunt thoracic trauma, pneumomediastinum has been reported in 2.6%–10% of the cases [13,14,16]. According to Mucart and associates, in the absence of aerodigestive tract injuries, pneumomediastinum associated with chest trauma is a benign condition and does not predict increased mortality [16]. In the current report, pneumomediastinum was found in six (5.6%) of the participants. However, it was found more in those cases died (50%), and the difference was highly significant (P-Value <0.001). Again, this could be explained by the bias created by the omitting chest CT scan in the alive patients as almost all of the dead patients (93.8%) underwent CT Scan [14].

Securing the airway is the first and the most crucial step in the management of any trauma patient. Endotracheal intubation is an imminent strategy when an airway is about to collapse. However, intubation and subsequent ICU admission are well-recognized to be the two predictors of mortality in chest trauma [11,14]. This correlation was confirmed by the current study (both P-values were <0.001).

## 5 Conclusion

The patients with bilateral thoracic trauma have comparable patterns of presentation, investigation of choices, strategies of management, predictors of the outcome, morbidity and mortality with unilateral chest trauma. The overall outcome depends on the type of trauma and severity of the insult.

## Ethical approval

Ethical approval was given by Ethical and Scientific Committee of Kurdistan Board for Medical Specialties. 28/10/2018. No. 545.

## Funding

None is found.

## Author contribution

Aram Baram: Surgeon supervising the management, revising the manuscript. Final approval of the manuscript.

Fahmi Hussien Kakamad: data collection, drafting the manuscript. Final approval of the manuscript.

## Conflicts of interest

None to be declared.

## Research registration unique identifying number (UIN)

ChiCTR1900021262 from Chinese Clinical Trial Registry.

## Guarantor

Fahmi Hussein Kakamad.
